# Alkali-metal cations steer the product selectivity of O_2_ reduction on M–N_4_ sites

**DOI:** 10.1093/nsr/nwaf201

**Published:** 2025-05-21

**Authors:** Yue Feng, Yu-Qi Wang, Zi-Cong Wang, Hong Li, Liang Ding, Jin-Song Hu, Li-Jun Wan, Dong Wang

**Affiliations:** CAS Key Laboratory of Molecular Nanostructure and Nanotechnology, Beijing National Laboratory for Molecular Science, Institute of Chemistry, Chinese Academy of Sciences, Beijing 100190, China; University of Chinese Academy of Sciences, Beijing 101408, China; CAS Key Laboratory of Molecular Nanostructure and Nanotechnology, Beijing National Laboratory for Molecular Science, Institute of Chemistry, Chinese Academy of Sciences, Beijing 100190, China; CAS Key Laboratory of Molecular Nanostructure and Nanotechnology, Beijing National Laboratory for Molecular Science, Institute of Chemistry, Chinese Academy of Sciences, Beijing 100190, China; University of Chinese Academy of Sciences, Beijing 101408, China; CAS Key Laboratory of Molecular Nanostructure and Nanotechnology, Beijing National Laboratory for Molecular Science, Institute of Chemistry, Chinese Academy of Sciences, Beijing 100190, China; University of Chinese Academy of Sciences, Beijing 101408, China; CAS Key Laboratory of Molecular Nanostructure and Nanotechnology, Beijing National Laboratory for Molecular Science, Institute of Chemistry, Chinese Academy of Sciences, Beijing 100190, China; University of Chinese Academy of Sciences, Beijing 101408, China; CAS Key Laboratory of Molecular Nanostructure and Nanotechnology, Beijing National Laboratory for Molecular Science, Institute of Chemistry, Chinese Academy of Sciences, Beijing 100190, China; University of Chinese Academy of Sciences, Beijing 101408, China; CAS Key Laboratory of Molecular Nanostructure and Nanotechnology, Beijing National Laboratory for Molecular Science, Institute of Chemistry, Chinese Academy of Sciences, Beijing 100190, China; University of Chinese Academy of Sciences, Beijing 101408, China; CAS Key Laboratory of Molecular Nanostructure and Nanotechnology, Beijing National Laboratory for Molecular Science, Institute of Chemistry, Chinese Academy of Sciences, Beijing 100190, China; University of Chinese Academy of Sciences, Beijing 101408, China

**Keywords:** electrochemical scanning tunneling microscopy, oxygen reduction reaction, single-atom catalysts, alkali-metal cations

## Abstract

The oxygen reduction reaction (ORR) to either H_2_O_2_ or H_2_O generation is important to meet diverse application demands. The product selectivity of the ORR is strongly correlated with the nature of the catalyst. We report herein that alkali-metal cations (AM^+^) can steer the product selectivity of the ORR catalysed on a molecular model catalyst with Co–N_4_ sites. The electron-transfer number of the ORR increases with Li^+^ ≈ Na^+^ < K^+^ < Rb^+^ < Cs^+^. A series of electrochemical measurements reveal the 2e^−^+2e^−^ ORR pathway in large AM^+^ electrolytes at neutral pH. *In situ* electrochemical scanning tunneling microscopy resolves the formation of high-contrast species in the cobalt octaethylporphine (CoOEP) monolayer on Au(111) in large AM^+^ electrolytes when the ORR occurs. The high-contrast species is assigned to the HO_2_^−^, as the 2e^−^ ORR product, adsorbed on CoOEP. Combined electrochemical scanning tunneling microscopy, electrochemical measurements and theoretical calculations reveal that large AM^+^ can stabilize HO_2_^−^ on CoOEP and promote its further reduction, which accounts for the AM^+^-dependent selectivity of the ORR. Revealing the unrecognized effect of AM^+^ on ORR selectivity opens up new avenues for modulating the distribution of ORR products by adjusting the electrolyte composition.

## Teaser

This study reveals that alkali metal cations stabilize reaction intermediates and steer oxygen reduction pathways, enabling selective hydrogen peroxide or water production through simple electrolyte tuning.

## INTRODUCTION

The oxygen reduction reaction (ORR) is one of the most widely investigated cathodic processes owing to its important role in energy conversion [1–4]. The ORR in neutral environments has received widespread attention due to its mild reaction conditions and promising applications in biological systems and seawater electrolysis [5,6]. Both 2e^−^ reduction producing H_2_O_2_ and 4e^−^ reduction producing H_2_O by using the ORR are valuable and highly pursued [7–10]. Generally, the product selectivity of the ORR is largely determined by the nature of the electrocatalytically active centers. For instance, 4e^−^ reduction is promoted on Pt, Fe–N_4_ and Mn–N_4_ catalytic sites [11–13] and 2e^−^ reduction dominates on Co–N_4_ sites [14,15]. To meet different practical demands, tremendous efforts have been devoted to developing specialized ORR catalysts with excellent activity and high selectivity through elaborately designed synthesis [7–10]. Recently, the influence of electrolyte components on the selectivity of small-molecule electrocatalysis has received increasing attention [16–18]. A versatile and cost-effective solution to steer the product selectivity of the ORR and to fit different application requirements is highly desirable.

Alkali-metal cations (AM^+^) are one of the most widely used supporting electrolytes. AM^+^ significantly affect the performance of many reduction reactions including the ORR [19–21], hydrogen evolution reaction [22,23] and CO_2_ reduction reaction [24–26]. The mechanism of the AM^+^ effect is a fundamental issue in electrochemistry that is currently receiving great attention. AM^+^ have been reported to modulate the potential profile in the electric double layer (EDL) and the surface charge density [24,27–32], buffer the interfacial pH and affect proton diffusion [33–36] and interact directly with reaction intermediates [37]. For example, it has been reported that hydrated AM^+^, especially Li^+^ and Na^+^, interact with the surface-adsorbed OH^−^ on Pt(111) and inhibit O_2_ from reaching the Pt surface, resulting in the lowered ORR activity [20]. M–N_4_ electrocatalysts are highly active towards the ORR and are promising alternatives to noble-metal materials [12–15]. The effect of AM^+^ on ORRs catalysed by M–N_4_ sites such as product selectivity has not yet been investigated. Metal porphyrins (MPors) and phthalocyanines are molecular models for practical M–N_4_ catalysts, which have been widely used in mechanistic studies of electrocatalysis. In this work, we used MPors as molecular models and explored the effect of AM^+^ on the ORR catalysed by M–N_4_ sites. Moreover, *in situ* observation of catalytic processes on surface active sites is valuable to provide visual evidence of the catalytic mechanism. Electrochemical scanning tunneling microscopy (EC–STM) allows imaging of surface catalysis at atomic and molecular levels under electrochemical conditions [38–44]. For instance, *in situ* EC–STM resolves the adsorption of O_2_ on FePc and reveals the catalytic conversion of O_2_ on FePc when the ORR occurs [45]. Moreover, it is reported that the ORR activity of Co–N_4_ sites is positively correlated with the Co–O_2_ binding strength, which provides *in situ* evidence of the scaling relationship [46].

Herein, we report that the product selectivity of ORRs catalysed by cobalt octaethylporphine (CoOEP) depends on the species of AM^+^ in the electrolyte. The electron-transfer number increases in the order of Li^+^ ≈ Na^+^ < K^+^ < Rb^+^ < Cs^+^. The O_2_ is reduced to H_2_O_2_ in small AM^+^ electrolytes and more completely to H_2_O in large AM^+^ electrolytes. The 2e^−^+2e^−^ ORR mechanism in large AM^+^ is revealed based on Damjanović kinetics. EC–STM is employed to *in situ* investigate the catalytic conversion of O_2_ on the CoOEP. High-contrast species are observed in large AM^+^ electrolytes when the ORR occurs and assigned to the 2e^−^ ORR product adsorbed on the CoOEP. Moreover, the electron-transfer number of the ORR is positively correlated with the surface coverage of the adsorbed 2e^−^ ORR product. The result shows that AM^+^ modulate the product selectivity of the ORR by regulating the stability of the adsorbed 2e^−^ ORR product. In addition to the previously established dependence of ORR selectivity on the nature of the catalyst, this study opens up a new avenue to steer ORR selectivity by modifying the composition of the electrolyte.

## RESULTS AND DISCUSSION

### Selectivity of ORR catalysed by the CoOEP in various AM^+^ electrolytes

**Figure 1.**
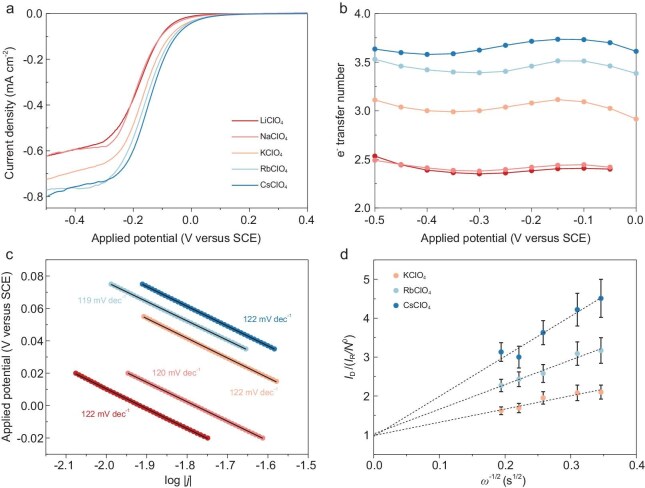
Electrochemical measurements of ORR in different AM^+^ electrolytes. (a and b) ORR catalysed by CoOEP in various AMClO_4_ electrolytes. (a) LSVs of ORR catalysed by CoOEP in O_2_-saturated 80 mM AMClO_4_ electrolytes (AM = Li, Na, K, Rb, Cs). (b) Electron-transfer number of ORR measured in (a). (c) Tafel plots of ORR measured in (a). (d) Analysis of Damjanović kinetics of ORR catalysed by CoOEP in O_2_-saturated 80 mM KClO_4_, RbClO_4_ and CsClO_4_ electrolytes.

Rotating ring-disk voltammetry is employed to study the effect of AM^+^ on the ORR performance catalysed by using CoOEP. The ORR is measured on the CoOEP-modified Au disk and the produced hydrogen peroxide is detected on the Pt ring. Figure 1a shows the linear-sweep voltammograms (LSVs) in O_2_-saturated AMClO_4_ electrolytes (AM = Li, Na, K, Rb and Cs). The reduction current is ascribed to the ORR and is not detected in Ar-saturated electrolytes. The onset potential for the ORR is more positive and the reduction current density is higher in larger AM^+^ electrolytes. Figure 1b shows the product selectivity of the ORR in different AM^+^ electrolytes. The electron-transfer number increases following the trend of Li^+^ ≈ Na^+^ < K^+^ < Rb^+^ < Cs^+^. It has been reported that cobalt porphyrins and phthalocyanines predominantly promote the 2e^−^ ORR [47]. The influence of AM^+^ on the product selectivity of ORRs catalysed by CoOEP suggests the noticeable role of large AM^+^ in facilitating the 4e^−^ ORR process.

Furthermore, the Tafel slope values of the ORR in different AM^+^ electrolytes are similar (∼120 mV/dec, Fig. 1c), suggesting that the rate-determining step (RDS) of the O_2_ reduction in all AM^+^ electrolytes is the first electron transfer. M–N_4_ sites can catalyse the reduction of O_2_ to H_2_O via either the direct 4e^−^ or the 2e^−^+2e^−^ ORR pathway. The cleavage of the M–O bond occurs after the four-electron transfer in the direct 4e^−^ pathway. In the 2e^−^+2e^−^ pathway, O_2_ is reduced via the 2e^−^ ORR to hydrogen peroxide, which is further reduced to achieve the four-electron transfer. To study the pathway of the nearly 4e^−^ O_2_ reduction in large AM^+^ electrolytes, we analysed the Damjanović kinetics of the ORR (Fig. S1) [48–50]. The rotating ring-disk voltammograms are measured on the CoOEP in O_2_-saturated CsClO_4_ electrolyte at various rates of electrode rotation (*ω*). As shown in Fig. 1d, *I*_D_/(*I*_R_/*N*^0^) (*I*_D_, *I*_R_ and *N*^0^ are the disk current, ring current and collection efficiency, respectively) measured at −0.2 V (vs. a saturated calomel electrode (SCE)) is plotted versus *ω*^−1/2^. The intercept is fitted to be 0.96 and represents 1+(2*k*_1_/*k*_2_), where *k*_1_ and *k*_2_ are the rate constants of the direct 4e^−^ ORR and 2e^−^ ORR, respectively. According to the Damjanović kinetics of the ORR [48–50], an intercept of ∼1 indicates that *k*_1_ is close to 0, showing that the direct 4e^−^ process barely occurs. Here, the intercept of 0.96 suggests that the ORR catalysed by CoOEP in the Cs^+^ electrolyte follows the 2e^−^+2e^−^ reduction. Similarly, the intercept is fitted to be 0.98 and 1.03 in K^+^ and Rb^+^ electrolytes, respectively (Fig. 1d). The electron-transfer number of the ORR positively correlates with the size of the AM^+^, showing that the reduction of the 2e^−^ ORR product is facilitated in large AM^+^ electrolytes, which is summarized as:


(1) \begin{eqnarray*}
&&{{\mathrm{O}}}_2 + 2{{\mathrm{e}}}^ - \to 2{{\mathrm{e}}}^ -\,\,{\mathrm{ORR\ product}} \\
&&\left( {{\mathrm{in\ all\ A}}{{\mathrm{M}}}^ + {\mathrm{electrolytes}}} \right)
\end{eqnarray*}

(2) \begin{eqnarray*}
&& 2{{\mathrm{e}}}^ -\,\, {\mathrm{ORR\ product}} + 2{{\mathrm{e}}}^ - \to 2{{\mathrm{H}}}_2{\mathrm{O}} \\
&& \left( {{\mathrm{in}}\, {{\mathrm{K}}}^ + ,{\mathrm{R}}{{\mathrm{b}}}^ + ,{\mathrm{and\ C}}{{\mathrm{s}}}^ + {\mathrm{electrolytes}}} \right)
\end{eqnarray*}

### EC–STM study of the ORR in large AM^+^ electrolytes

**Figure 2.**
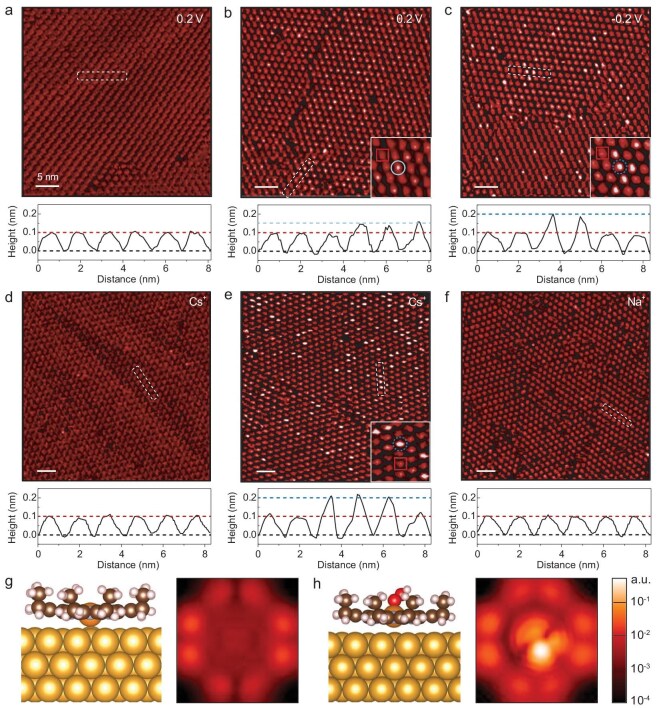
EC–STM investigation of ORR in large AM^+^ electrolytes. (a) EC–STM image of CoOEP monolayer in Ar-saturated 80 mM CsClO_4_ electrolyte at 0.2 V. (b and c) EC–STM images of CoOEP monolayer in O_2_-saturated 80 mM CsClO_4_ electrolyte at (b) 0.2 V and (c) −0.2 V. (d–f) EC–STM images of CoOEP monolayer in electrolyte containing 3 mM of hydrogen peroxide at −0.2 V. (d) CoOEP monolayer in Ar-saturated 80 mM CsClO_4_ electrolyte. (e) CoOEP monolayer in Ar-saturated 80 mM Cs^+^ electrolyte with pH 12.5. (f) CoOEP monolayer in Ar-saturated 80 mM Na^+^ electrolyte with pH 12.5. Electrolytes used in (e) and (f) are prepared with AMClO_4_ and AMOH. Cross section corresponding to the dashed box. Scale bar represents 5 nm. (g and h) Calculated molecular structures and simulated EC–STM images of (g) CoOEP and (h) HO_2_^−^–CoOEP.

EC–STM was employed to investigate the surface species in the self-assembled CoOEP monolayer on Au(111) prior to and during the ORR. As shown in Fig. 2a, in the Ar-saturated CsClO_4_ electrolyte, the CoOEP molecules (as marked by the solid square in Fig. 2) appear as bright spots with an apparent height of ∼0.1 nm and they barely undergo any morphological transformation in the potential range of 0.2 to −0.2 V (Fig. S2). In the O_2_-saturated CsClO_4_ electrolyte, species with an apparent height of ∼0.15 nm (as marked by the solid circle in Fig. 2b) appears in the CoOEP monolayer at 0.2 V, which is assigned to the O_2_ adsorption on the CoOEP [46]. At −0.2 V, the high-contrast species (i.e. surface species with relatively high apparent height) [51,52] with an apparent height of ∼0.2 nm (as marked by the dotted circle in Fig. 2c) is observed in the CoOEP monolayer. The high-contrast species is not observed in the Ar-saturated electrolyte and at 0.2 V (i.e. when the ORR does not occur) in the O_2_-saturated electrolyte. Moreover, the high-contrast species is not observed in the O_2_-saturated AM^+^-free and small AM^+^ electrolytes when the ORR occurs (see above) [46]. These results suggest that the high-contrast species is related to the O_2_ reduction and indicate the essential role of large AM^+^ in promoting the formation of high-contrast species. It is reported that the 2e^−^ ORR product readily desorbs from Co–N_4_ sites due to the relatively weak Co–O bond, resulting in the 2e^−^ ORR pathway [47]. Enhancing the stability of the 2e^−^ ORR product adsorbed on the Co–N_4_ sites is important for the 4e^−^ ORR pathway. Here, the high-contrast species generated via the ORR is more stable in large AM^+^ electrolytes. Additionally, electrochemical measurements (Fig. 1) have shown that, in large AM^+^ electrolytes, the reduction of the 2e^−^ ORR product is facilitated and the 4e^−^ ORR pathway is favored. The previous studies and our results inspire us to further investigate the correlation between the high-contrast species and the 2e^−^ ORR product [24,47].

To assign the high-contrast species, EC–STM is conducted to observe the CoOEP monolayer with H_2_O_2_ in the electrolyte. As shown in Fig. 2d and Fig. S3, in the Ar-saturated CsClO_4_ electrolyte with 3 mM H_2_O_2_, the surface is fully covered by CoOEP molecules with an apparent height of ∼0.1 nm. The high-contrast species barely appears in the monolayer, demonstrating that the high-contrast species observed during the ORR in the Cs^+^ electrolyte does not originate from the H_2_O_2_ adsorption. It is reported that the O_2_ reduction leads to the elevated pH value in the vicinity of the electrode surface [53]. The elevated pH value affects the acid–base equilibrium of the hydrogen peroxide and favors the presence of the HO_2_^−^ form. These results inspire us to investigate the binding of HO_2_^−^ on CoOEP and its effect on the apparent height of the surface species [54–56]. As shown in Fig. 2e, the CoOEP monolayer is observed in the Ar-saturated Cs^+^ electrolyte with pH 12.5 containing 3 mM of hydrogen peroxide. The pKa for H_2_O_2_ is 11.6 and the hydrogen peroxide in the electrolyte exists mainly in the form of HO_2_^−^ with pH 12.5 [55]. Surface species with an apparent height of ∼0.2 nm is observed in the monolayer (Fig. 2e), which is assigned to the adsorbed HO_2_^−^ on the CoOEP. The assignment is further confirmed by the absence of the 0.2-nm-height species in the HO_2_^−^-free Cs^+^ electrolyte with pH 12.5 (Fig. S4). Moreover, the adsorption of HO_2_^−^ on the CoOEP is barely observed in the Na^+^ electrolyte with pH 12.5 containing 3 mM of hydrogen peroxide (Fig. 2f), which suggests that the adsorbed HO_2_^−^ is stabilized by large AM^+^. The adsorbed HO_2_^−^ and the high-contrast species observed in the O_2_-saturated CsClO_4_ electrolyte when the ORR occurs not only exhibit the same contrast, but their formation also shares consistent AM^+^ dependence. Thus, the high-contrast species is assigned to the adsorbed HO_2_^−^ on the CoOEP. Furthermore, EC–STM is employed to observe the CoOEP monolayer in O_2_-saturated various AM⁺ electrolytes at pH 1.5 (Figs S5 and S6). The 2e⁻ ORR product exists in the form of H_2_O_2_ due to the acid–base equilibrium of hydrogen peroxide in acidic environments. Under ORR conditions, the high-contrast species is not observed in all AM⁺ electrolytes. These results are consistent with the assignment of the high-contrast species as the adsorbed HO_2_^−^ on the CoOEP. Overall, EC–STM results demonstrate that adsorbed HO_2_^−^ as the 2e^−^ ORR product is stabilized by large AM^+^ in the electrolyte.

The assignment of surface species is further confirmed by using theoretical calculations based on density functional theory (DFT). The calculated molecular structures of CoOEP and HO_2_^−^–CoOEP are shown in Fig. 2 and Fig. S7. In the simulated EC–STM images (Fig. 2g and h), HO_2_^−^–CoOEP shows significantly more enhanced contrast than CoOEP, which is in good agreement with the experimental observation.

**Figure 3.**
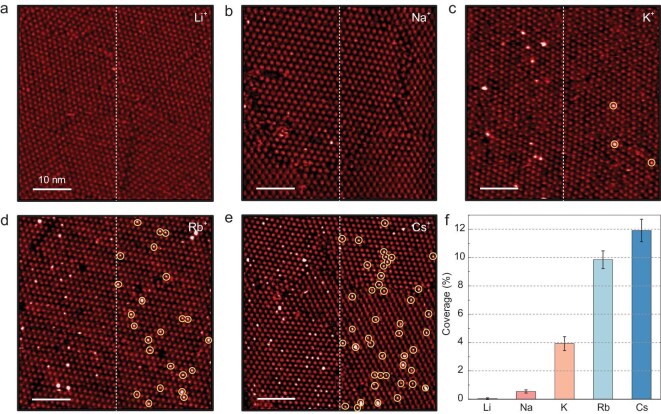
AM^+^ effect on HO_2_^−^ binding to CoOEP. (a–e) EC–STM images of CoOEP monolayer in O_2_-saturated 80 mM AMClO_4_ electrolytes at −0.2 V. Scale bar represents 10 nm. (f) Statistics of surface HO_2_^−^–CoOEP coverage measured in (a–e).

Furthermore, EC–STM is conducted to investigate the stabilizing effect of all AM^+^ on HO_2_^−^ binding to CoOEP when the ORR occurs. As shown in Fig. 3a and b, the adsorbed HO_2_^−^ is barely observed in the Li^+^ and Na^+^ electrolytes. In the K^+^ electrolyte (Fig. 3c), a noticeable amount of adsorbed HO_2_^−^ is observed with a surface coverage of ∼4%. The HO_2_^−^ coverage increases to ∼10% in the Rb^+^ electrolyte (Fig. 3d) and ∼12% in the Cs^+^ electrolyte (Fig. 3e). The statistical results quantitatively demonstrate that the surface HO_2_^−^ coverage is positively correlated with the size of the AM^+^ in the electrolyte (Fig. 3f). Additionally, this result is in good agreement with the presence and absence of adsorbed HO_2_^−^ in the Cs^+^ and Na^+^ electrolytes (with HO_2_^−^ in the electrolyte), respectively (Fig. 2e and f). It is known that the surface coverage of adsorbates is positively correlated with the equilibrium constant or adsorption energy [37,46]. The EC–STM results suggest that the stabilizing effect of AM^+^ on the adsorbed HO_2_^−^ increases in the order of Li^+^ ≈ Na^+^ < K^+^ < Rb^+^ < Cs^+^.

It is reported that AM^+^ accumulates in the EDL during cathodic polarization [57,58] in the order of Li^+^ < Na^+^ < K^+^ < Rb^+^ < Cs^+^. The AM^+^ in EDL undergo partial dehydration [59] and can stabilize surface-adsorbed species such as reactants, intermediates and products [24,37]. For instance, it has been proposed that the dipole field generated by AM^+^ in the outer Helmholtz plane can stabilize CO during CO_2_ reduction [24]. Here, we investigated the effect of the electric field on the stability of HO_2_^−^–CoOEP. As the electric field strength increases, the free-energy change of HO_2_^−^–CoOEP (compared with that without the electric field) gradually increases (Figs S8 and S9). We propose that a similar dipole field effect may account for the stabilization of HO_2_^−^–CoOEP in large AM^+^ electrolytes.

### Stabilization of the 2e^−^ ORR product results in an increased electron-transfer number

**Figure 4.**
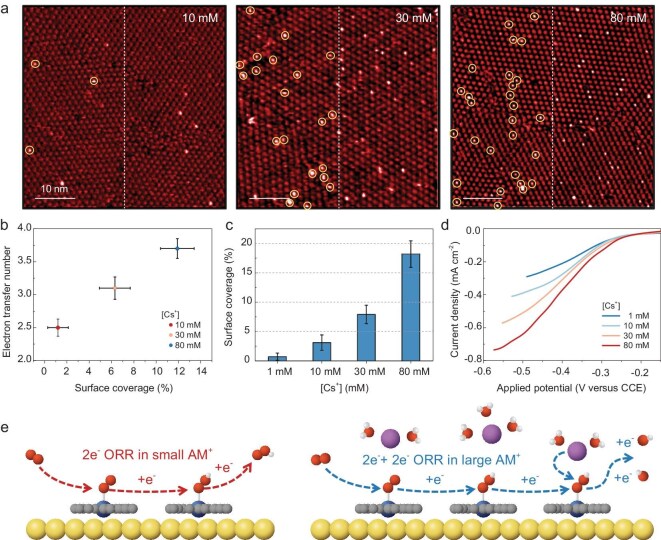
The 2e^−^+2e^−^ ORR process in large AM^+^ electrolytes. (a) EC–STM images of CoOEP monolayer in O_2_-saturated 80 mM AMClO_4_ electrolytes with [Cs^+^] of 10, 30 and 80 mM at −0.2 V. Electrolytes were prepared by using LiClO_4_ and CsClO_4_. Scale bars represent 10 nm. (b) Correlation between the surface HO_2_^−^–CoOEP coverage measured in (a) and the electron-transfer number of the ORR at −0.2 V in electrolytes with various [Cs^+^]. (c) Surface HO_2_^−^ coverage in Ar-saturated electrolytes containing 3 mM of hydrogen peroxide and various [Cs^+^]. (d) LSVs of HO_2_^−^ reduction on CoOEP in Ar-saturated electrolytes with 3 mM of hydrogen peroxide and various Cs^+^ concentrations. Electrolytes for measurements in (c) and (d) were prepared by using AMClO_4_ and AMOH to give a pH value of 12.5. Li^+^ was used to maintain the AM^+^ concentration at 80 mM. (e) Scheme illustrating the 2e^−^ ORR process in small AM^+^ (Li^+^ and Na^+^) electrolytes and the 2e^−^+2e^−^ ORR process in large AM^+^ (K^+^, Rb^+^ and Cs^+^) electrolytes.

Furthermore, we investigated the correlation between the surface HO_2_^−^ coverage and the electron-transfer number of the ORR in O_2_-saturated electrolytes with Cs^+^ concentrations ([Cs^+^]) of 10, 30 and 80 mM. As the effect of Li^+^ on ORR selectivity (Fig. 1) and HO_2_^−^ adsorption (Fig. 3) is negligible, the electrolytes are prepared here by using CsClO_4_ and LiClO_4_ to keep [AMClO_4_] at 80 mM. As shown in Fig. 4a, the surface coverage of HO_2_^−^ at −0.2 V is 1.2% ([Cs^+^] = 10 mM) ˂ 6.3% ([Cs^+^] = 30 mM) ˂ 11.9% ([Cs^+^] = 80 mM). In addition, the electron-transfer number of the ORR at −0.2 V is 2.5 ([Cs^+^] = 10 mM) ˂ 3.1 ([Cs^+^] = 30 mM) ˂ 3.7 ([Cs^+^] = 80 mM) (Fig. 4b). The statistical result suggests the positive correlation between the electron-transfer number of the ORR and the surface HO_2_^−^ coverage (Fig. 4b).

Moreover, to explore the effect of stabilized HO_2_^−^ adsorption on HO_2_^−^ reduction, we measured the HO_2_^−^ reduction current in Ar-saturated electrolytes containing 3 mM of hydrogen peroxide and various [Cs^+^]. The electrolyte is prepared by using AMClO_4_ and AMOH to allow pH 12.5. Li^+^ is used to maintain the AM^+^ concentration at 80 mM. Electrochemical measurements suggest the positive correlation between the onset potential of the HO_2_^−^ reduction and [Cs⁺], indicating the involvement of [Cs⁺] in HO_2_^−^ reduction. We then investigated the influence of Cs⁺ on the surface coverage of HO_2_^−^, as well as the relationship between the surface HO_2_^−^ coverage and the onset potential of the HO_2_^−^ reduction. In EC–STM images (Fig. S10), the surface HO_2_^−^ coverage in the CoOEP monolayer is ∼0.7% ([Cs^+^] = 1 mM) ˂ 3.1% ([Cs^+^] = 10 mM) ˂ 7.9% ([Cs^+^] = 30 mM) ˂ 18.2% ([Cs^+^] = 80 mM) (Fig. 4c). Correlative EC–STM and LSVs (Fig. S11) demonstrate that the HO_2_^−^ reduction current is positively correlated with the surface HO_2_^−^ coverage. To further probe the interfacial dynamics, we plotted the LSVs on the HO_2_^−^-coverage-corrected electrode (CCE) scale [60], which is defined as:


(3) \begin{eqnarray*}
{E}_{\rm CCE} = {E}_{\rm SCE} - 0.059 \times {\mathrm{log}}\theta ,
\end{eqnarray*}

where *θ* represents the surface coverage of adsorbed HO_2_^−^ on the CoOEP. When the surface coverage of HO_2_^−^ is varied, the onset potentials for HO_2_^−^ reduction vs SCE are distinct (Fig. S11) yet they become unified when referenced to the CCE (Fig. 4d), suggesting that the adsorbed HO_2_^−^ acts as the reactant in the RDS of the HO_2_^−^ reduction (Fig. S11). The HO_2_^−^ reduction is facilitated by the increased coverage of adsorbed HO_2_^−^ under the stabilization effect of large AM^+^. In addition, the high-contrast species was shown previously to be the 2e^−^ ORR product and herein to be the reaction substrate for HO_2_^−^ reduction, which further confirms that the high-contrast species is adsorbed HO_2_^−^ on the CoOEP. Briefly, the large AM^+^ stabilize the adsorbed HO_2_^−^ and promote the reduction of HO_2_^−^ to H_2_O, thereby facilitating the 2e^−^+2e^−^ ORR.

It is reported that the Co–O bond is relatively weak compared with Fe–O and Mn–O bonds, leading to the facile desorption of the 2e^−^ ORR product (Fig. S9) and consequently favoring the 2e^−^ ORR (in contrast to FeN_4_ and MnN_4_ sites, which promote the 4e^−^ ORR) [47]. Here, we show that the surface coverage of adsorbed HO_2_^−^ is positively correlated with the size of AM^+^ and the concentration of large AM^+^. These results suggest that, in small AM^+^ electrolytes, the 2e^−^ ORR product generated on CoN_4_ sites is barely stabilized by AM^+^ and readily desorbs, resulting in predominant 2e^−^ ORR selectivity. In contrast, large AM^+^ stabilize the adsorbed 2e^−^ ORR product on CoN_4_ sites and facilitate its further reduction, thereby enabling the nearly 4e^−^ O_2_ reduction (Fig. 4e).

### Beyond model electrocatalysts

Furthermore, the effect of AM^+^ on ORR selectivity was measured on the practical Co–N_4_ electrocatalyst. Here, we examined the ORR catalysed by COF-366-Co, which is an accessible and widely investigated covalent organic framework (COF) constructed from CoPor (Fig. S12) [61,62], in different AM^+^ electrolytes. The O_2_ reduction current commences at ∼0 V in LSVs measured on the COF-366-Co-coated electrode (Fig. S13). The electron-transfer number of the ORR in different AM^+^ electrolytes at −0.35 V increases in the order of Li^+^ (2.52) < Na^+^ (2.57) < K^+^ (3.16) < Rb^+^ (3.43) < Cs^+^ (3.58). On practical electrocatalysts with Co–N_4_ sites as active centers, the electron-transfer number of the ORR is positively correlated with the size of the AM^+^ in the electrolyte, suggesting that the AM^+^ effect revealed on model catalytic sites can be extended to practical electrocatalysts. Moreover, COF-366-Co represents a series of electrocatalysts with high structural designability [63–65]. The electronic properties of active sites can be tuned by modifying the molecular building blocks [66–69]. Structural engineering and functional group modification endow these catalysts with synergistic effects [70,71]. Notably, the active site structures are similar to metalloporphyrin, ensuring the generality of the strategy revealed in molecular models for modulating the product distribution by adjusting the electrolyte composition.

## CONCLUSION

In summary, through correlative electrochemical measurements and *in situ* EC–STM, we found that AM^+^ steer the product selectivity of ORRs catalysed by Co–N_4_ sites. Rotating ring-disk voltammetry reveals that the electron-transfer number of the ORR increases in the order of Li^+^ ≈ Na^+^ < K^+^ < Rb^+^ < Cs^+^. Analysis of the Damjanović kinetics of the ORR in large AM^+^ electrolytes reveals the 2e^−^+2e^−^ ORR pathway. *In situ* EC–STM resolves HO_2_^−^ binding on CoOEP in large AM^+^ electrolytes when the ORR occurs. The surface CoOEP–HO_2_^−^ coverage is higher in larger AM^+^ electrolytes. Moreover, the electron-transfer number of the ORR is positively correlated with the surface coverage of the adsorbed HO_2_^−^. A series of voltammograms suggest that the HO_2_^−^ reduction is promoted in large AM^+^ electrolytes, facilitating the 2e^−^+2e^−^ ORR process. Conclusively, the stabilization and further reduction of adsorbed HO_2_^−^ on CoOEP is promoted by large AM^+^, resulting in the increased electron-transfer number of the ORR. This work reveals unrecognized effects of AM^+^ on the product selectivity of ORRs catalysed by Co–N_4_ sites and the mechanisms involved. The demonstration of the AM^+^ effect provides a promising approach to modulate ORR selectivity to meet diverse application demands by adjusting the electrolyte composition rather than replacing the catalyst.

## METHODS

### Chemicals and materials

The CoOEP was from Sigma-Aldrich (98%). 5,10,15,20-Tetrakis(4-aminophenyl)porphyrin cobalt (CoTAPP) was from J&K (95%). CoOEP and CoTAPP were utilized without further purification. 1,4-Benzenedicarboxaldehyde (BDA) was from J&K (99%). *N,N*-Dimethylformamide (DMF) was from Sigma-Aldrich. H_2_O_2_ was from Sigma-Aldrich (70%). HClO_4_ was from ALDRICH (70%, purity > 99.999%). LiClO_4_ was from Sigma-Aldrich (purity > 99.99%). LiOH was from J&K (purity > 99%). NaClO_4_ was from ACROS (purity > 99%). NaOH was from MACKLIN (purity > 99.9%). KClO_4_ was from ALDRICH (purity > 99.99%). KOH was from J&K (purity > 90%). Rb_2_CO_3_ was from MACKLIN (purity > 99.9%). RbClO_4_ electrolyte was prepared by using Rb_2_CO_3_ and HClO_4_. CsClO_4_ was from Thermo Scientific (reagent grade). CsOH was from MACKLIN (purity > 99.9%). Milli-Q water (18.2 MΩ·cm, TOC < 4 ppb) was used throughout the investigation.

The COF-366-Co was prepared according to previous reports [61,62]. CoTAPP (18 mg, 0.025 mmol), BDA (6.7 mg, 0.05 mmol), 1,2-dichlorobenzene (1 mL), *n*-butanol (1 mL) and 6 M aqueous acetic acid (0.2 mL) were added in a 5-mL Pyrex tube. The tube was sonicated for 15 minutes and then flash-frozen at 77 K (liquid N_2_ bath). After three freeze–pump–thaw cycles, the tube was evacuated to an internal pressure of 50 mTorr and flame-sealed. After heating at 120°C for 72 h, a dark purple precipitate was produced at the bottom of the tube. The precipitate, separated by filtration, was transferred to a Soxhlet extractor and washed thoroughly with tetrahydrofuran (THF) (24 h) and acetone (24 h). The material was then dried in a vacuum oven at 80°C for 12 h. Then, 1 mg of COF-366-Co was dispersed in 1 mL of solution (water:isopropanol:5 wt% Nafion solution in the ratio of 10 : 10 : 0.1 by volume) and sonicated to form the mixture. Subsequently, 1 mg of XC-72 carbon black was added to the mixture. The mixture was sonicated for 1 h to yield the homogeneous ink, after which 25 μL of catalyst ink was dropwise coated onto the Au disk of the rotating ring-disk electrode as the working electrode (loading: 0.2 mg cm^−2^).

Powder X-ray diffraction patterns were recorded on a PANalytical Empyrean Diffractometer operating at 40 kV and 40 mA using Cu Kα radiation (*λ* = 1.5416 Å) at ambient temperature in the range of 2°–30° at 3.5°/min.

### EC–STM measurements

All EC–STM images were collected by using the NanoScope E scanning tunneling microscope (Bruker, Inc.). Tungsten wire (Alfa Aesar, 0.25 mm in diameter) was electrochemically etched (0.6 M KOH, 20 V DC) and coated with nail polish to prepare the EC–STM tips. The Au(111) single crystal prepared by using the Clavilier method was used as working electrode [72]. The Au(111) electrode was annealed in a hydrogen–oxygen flame before each experiment. The self-assembled CoOEP monolayer was prepared by immersing the Au(111) electrode in the DMF solution of the CoOEP. The working electrode was transferred into the electrochemical cell with two platinum wires as the reference electrode and counter electrode.

### Theoretical calculations

All DFT calculations were performed by using the projector augmented wave method. The electronic exchange and correlation were described by using the Perdew–Burke–Ernzerhof functional with the generalized gradient approximation [73]. The cut-off of the plane wave was set as 600 eV. The criteria of force convergence were <0.02 eV/Å for geometry optimization [74].

### Electrochemical measurements

The electrochemical measurements were conducted on an RRDE-3A (ALS, Japan) and an Autolab PGSTAT302N (Metrohm, Netherlands) electrochemical workstation. The LSVs were measured on the CoOEP-modified (as well as COF-366-Co-coated) Au disk electrode (4 mm in diameter). The ring electrode of the rotating ring-disk electrode was Pt. Prior to electrochemical measurements, the rotating-disk electrode and the rotating ring-disk electrode were polished with 0.3 and 0.05 μm of alumina slurries and then cleaned and subsequently sonicated in Milli-Q water and 2-propanol to a mirror-finish state. The Pt ring was then electrochemically cleaned. An SCE and a Pt wire were used as the reference and counter electrodes, respectively. All electrode potentials were reported with respect to the SCE. The LSVs were recorded at a scan rate of 10 mV s^−1^ without iR-compensation. Except for specific indications, the rate of electrode rotation was 100 rpm. The electron-transfer number of the ORR was calculated as *n* = 4*I*_D_/[(*I*_R_/*N*^0^)+*I*_D_].

## Supplementary Material

nwaf201_Supplemental_File
