# Supplementary material for: Alkali-metal cations steer the product selectivity of O2 reduction on M–N4 sites
Source: Natl Sci Rev. 2025 May 21;12(7):nwaf201. doi: 10.1093/nsr/nwaf201 (PMC12202207; doi:10.1093/nsr/nwaf201)
Supplement: nwaf201_Supplemental_File [file nwaf201_supplemental_file.pdf]

Supplementary information

Alkali metal cations steer the product selectivity of O_2 reduction on M-N_4 sites

Yue Feng[1,2,†], Yu-Qi Wang[1,†], Zi-Cong Wang[1,2], Hong Li[1,2], Liang Ding[1,2], Jin-Song Hu[1,2], Li-Jun Wan[1,2] and Dong Wang[1,2,*]

[1]CAS Key Laboratory of Molecular Nanostructure and Nanotechnology, Beijing National Laboratory for Molecular Science, Institute of Chemistry, Chinese Academy of Sciences, Beijing 100190, China

[2]University of Chinese Academy of Sciences, Beijing 101408, China

[*]Corresponding author. E-mail: wangd@iccas.ac.cn (D. Wang)

[†]Equally contributed to this work.

Contents:

Supplementary Figures S1 to S13

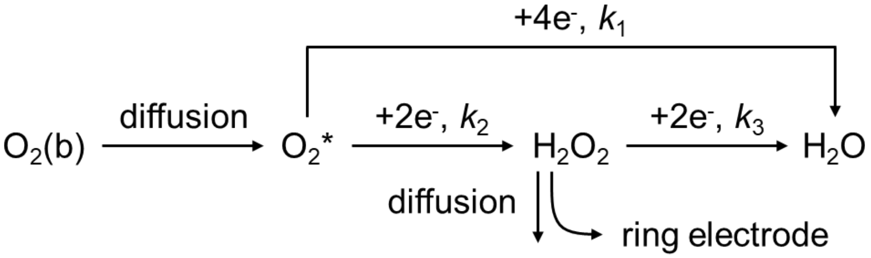

Figure S1. Scheme shows the Damjanović kinetics of ORR. Indices b designate the bulk of electrolytes. Indices * designate the vicinity of the disk electrode.

According to previous reports [1–3], the disk and ring current and the rate of electrode rotation satisfy the following equation:

$$\frac{I_D}{I_R/N^0}=\left(1+\frac{2k_1}{k_2}\right)+2k_3\left(1+\frac{k_1}{k_2}\right)/\gamma\sqrt{\omega} \qquad \text{(S1)}$$

Where I_D is the disk current, I_R is the ring current, N^0 is the collection efficiency, ω is rate of electrode rotation, and $\gamma=0.62D_{H_2O_2}^{2/3}\upsilon^{-1/6}$ ($D_{H_2O_2}$ is the diffusion coefficients of H_2O_2 and υ is the kinematic viscosity of the solution).

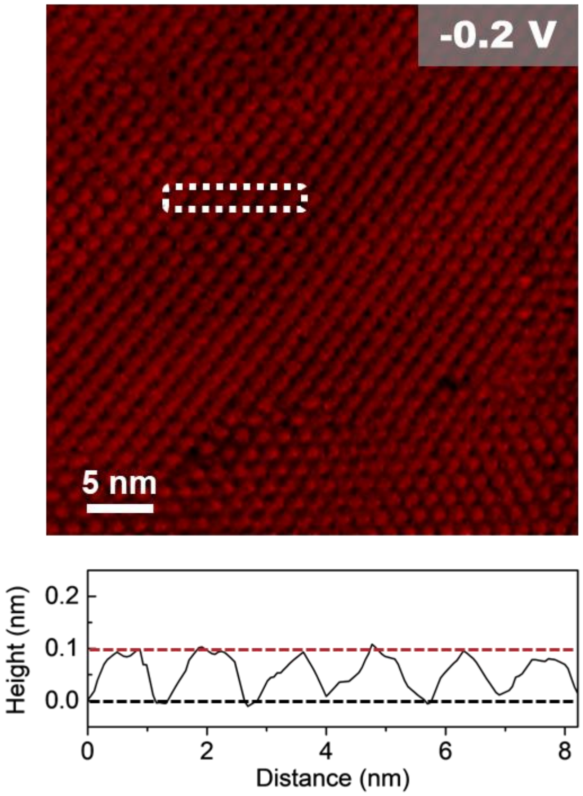

Figure S2. EC-STM image and corresponding section analysis of the CoOEP monolayer in Ar-saturated 80 mM $CsClO_4$ electrolyte at -0.2 V.

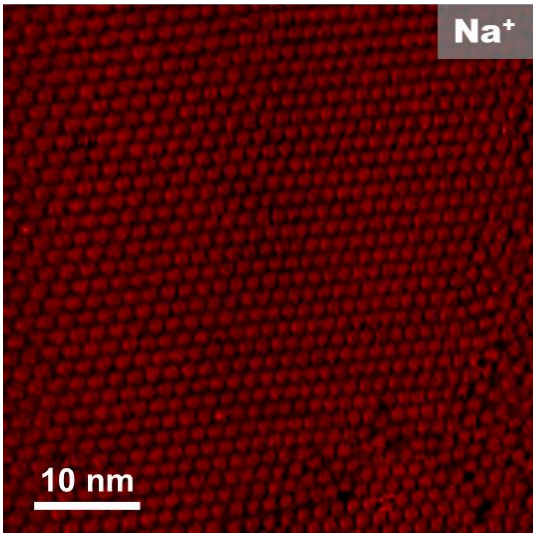

Figure S3. EC-STM image of the CoOEP monolayer in Ar-saturated 80 mM $NaClO_4$ electrolyte with 3 mM hydrogen peroxide at -0.2 V.

EC-STM is conducted to investigate the adsorption of hydrogen peroxide on CoOEP. In the potential range of -0.2 V to 0.2 V, high contrast species is not observed in 80 mM $NaClO_4$ electrolyte, 80 mM $CsClO_4$ electrolyte, and 80 mM Na^+ electrolyte (pH=12.5), while high contrast species is observed in 80 mM Cs^+ electrolyte (pH=12.5).

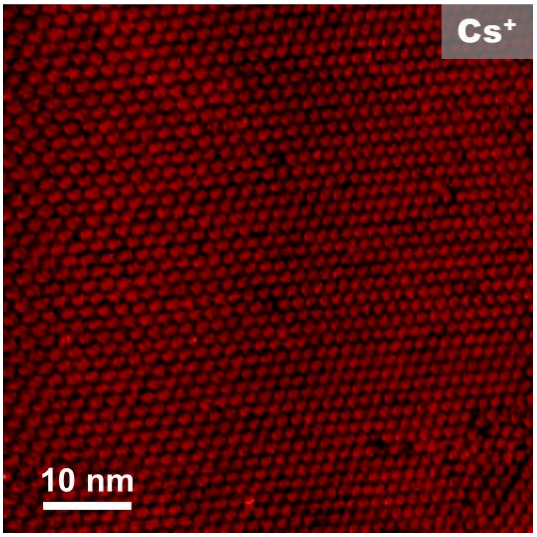

Figure S4. EC-STM image of the CoOEP monolayer in Ar-saturated 80 mM Cs^{+} electrolyte with pH=12.5 at -0.2 V. The electrolyte is prepared with $CsClO_4$ and CsOH.

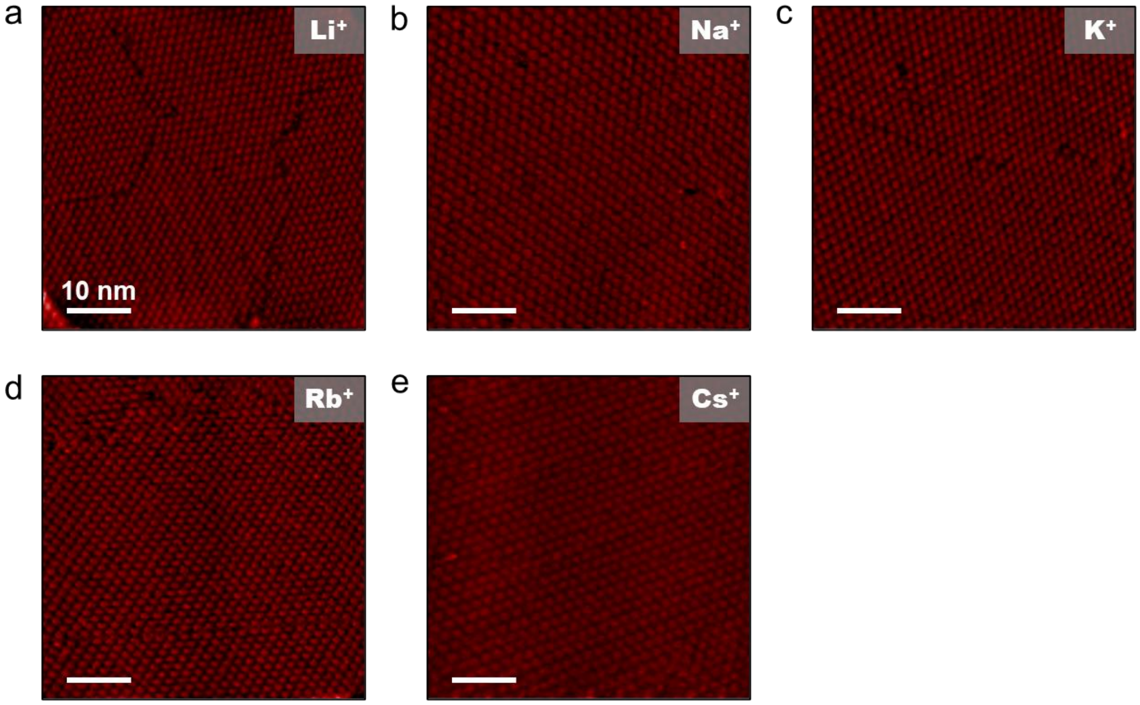

Figure S5. EC-STM images of the CoOEP monolayer in O_2-saturated AM^+ electrolytes with pH=1.5 at 0.15 V. The electrolyte is prepared with 30 mM $HClO_4$ and 50 mM $AMClO_4$.

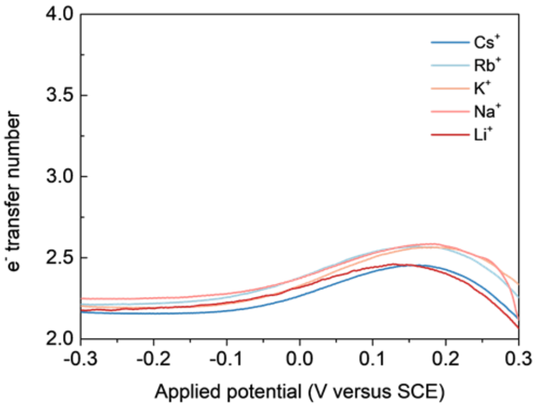

Figure S6. Electron transfer number of ORR catalyzed by CoOEP in O_2-saturated different AM^+ electrolytes. The electrolyte is prepared with 30 mM $HClO_4$ and 50 mM $AMClO_4$.

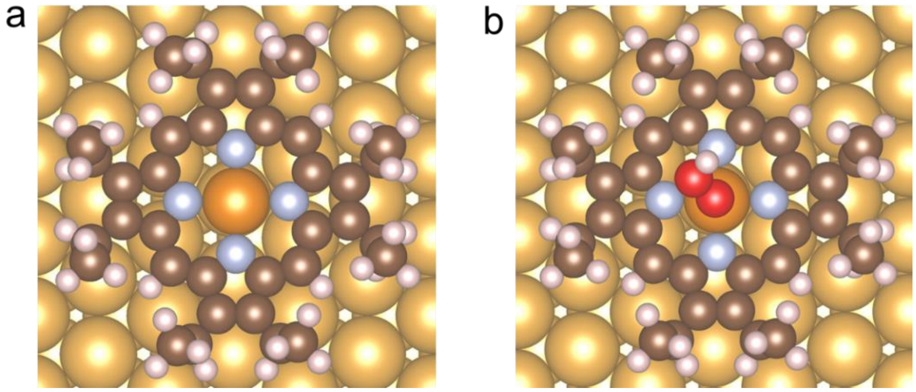

Figure S7. Geometry optimization of (a) CoOEP and (b) HO_2^--CoOEP.

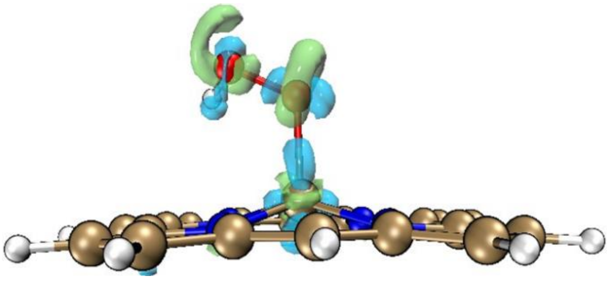

Figure S8. Charge density differences of HO_2^--CoOEP with a -0.51 V/Å electric field. Green and blue indicates gaining and losing electrons, respectively.

The charge distribution of HO_2^--CoOEP and the effect of AM^+ field on the stability of HO_2^--CoOEP are investigated. As shown in Figure S8, with a -0.51 V/Å electric field, the dipole moment of the Co-O bond increases and is oriented parallel to the direction of the AM^+ field.

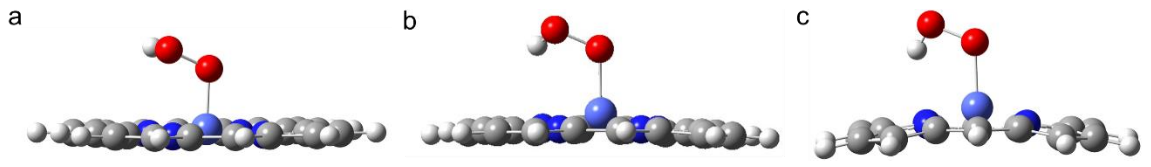

Figure S9. (a) Geometry optimization of HOO-CoOEP. (b) Geometry optimization of HO_2^--CoOEP without electric field. The dipole moment of HO_2^--CoOEP is calculated to be -1.08 eÅ. (c) Geometry optimization of HO_2^--CoOEP with a electric field of -0.51 V/Å.

As shown in Figure S9, the Co-O bond length increases from 1.8 Å in HOO-CoOEP to 1.9 Å in HO_2^--CoOEP, suggesting that the HO_2^- generated during ORR is easily desorbed. Moreover, the O-O bond length increases from 1.38 Å in HOO-CoOEP to 1.44 Å in HO_2^--CoOEP. The increase in the O-O bond length favors O-O breaking in the further reduction of the 2e$^-$ ORR products to H_2O.

Based on previous studies [4], we conducted comparative calculations of HO_2^--CoOEP without electric field and with electric fields of -0.26 and -0.51 V/Å. With increasing electric field strength, the free energy change of HO_2^--CoOEP (relative to that without the electric field) becomes increasingly negative, reaching -0.37 eV at -0.26 V/Å and -0.90 eV at -0.51 V/Å. These results suggest that the dipole field generated by AM^+ effectively enhances the stability of HO_2^--CoOEP.

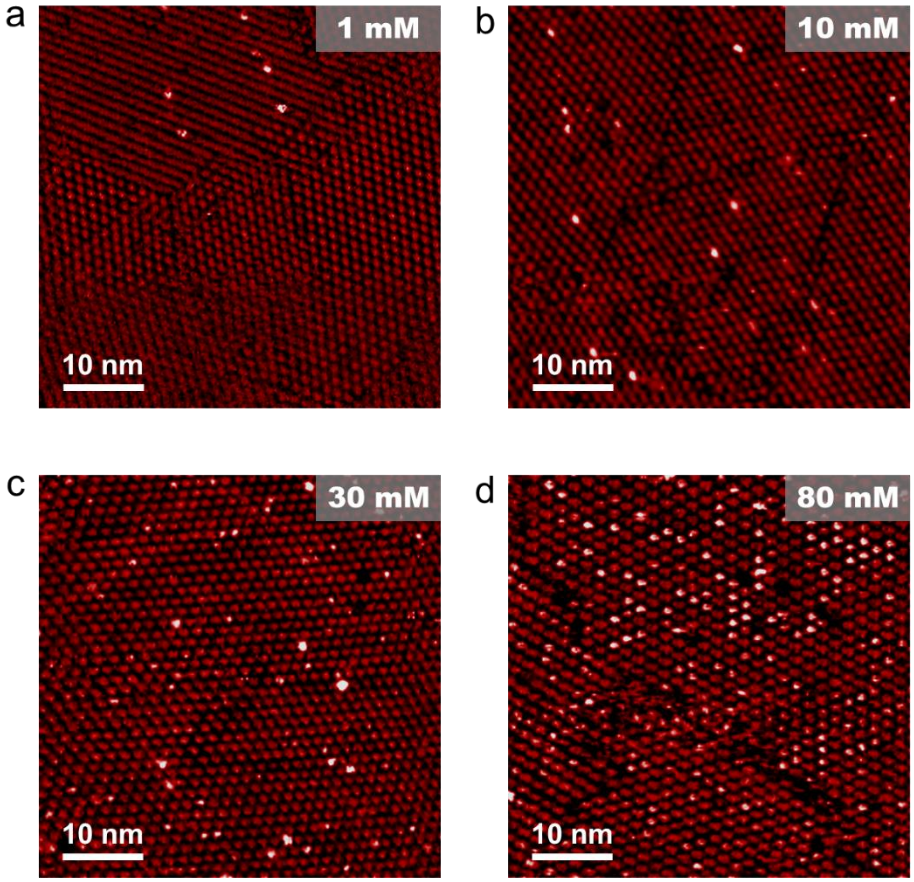

Figure S10. EC-STM images of the CoOEP monolayer in Ar-saturated electrolyte (pH=12.5) with 3 mM hydrogen peroxide and various Cs^+ concentration at -0.2 V. Li^+ is used to maintain the AM^+ concentration at 80 mM. The electrolyte is prepared with $AMClO_4$ and AMOH. The concentration of Cs^+ is (a) 1 mM, (b) 10 mM, (c) 30 mM, and (d) 80 mM.

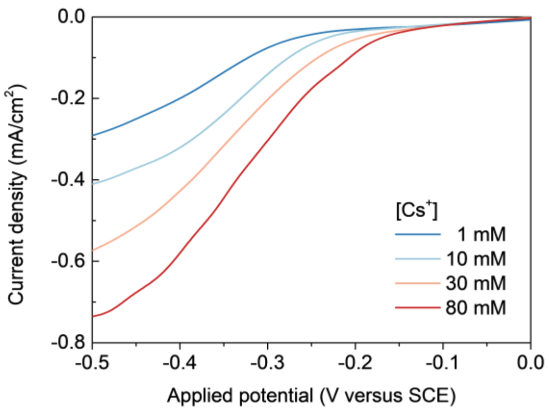

Figure S11. LSVs of HO_2^- reduction on CoOEP in Ar-saturated electrolytes (pH=12.5) with 3 mM hydrogen peroxide and various Cs^+ concentration. Li^+ is used to maintain the AM^+ concentration at 80 mM. The electrolyte is prepared with $AMClO_4$ and AMOH.

Electrochemical measurements (Figures 4d and S11) demonstrate that the onset potential for HO_2^- reduction remains consistent against CCE ($E_{CCE}=E_{SCE}-0.059\times\log\theta$) across varying surface coverages of HO_2^-. This indicates that the reduction potential E_{SCE} contains only one term related to θ, which is $-0.059\times\log(1/\theta)$. According to the Nernst equation, the onset potential is determined by the concentrations of the reactants and products involved in the rate-determining step (RDS). The term $-0.059\times\log(1/\theta)$ suggests that the adsorbed HO_2^- acts as the reactant in the RDS.

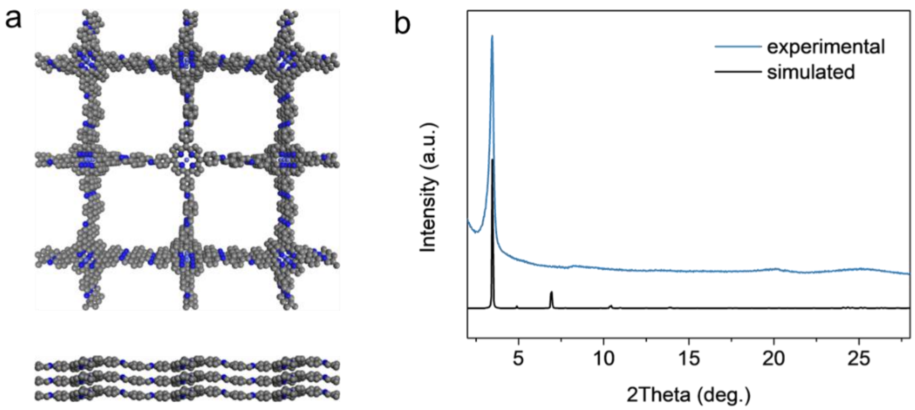

Figure S12. Characterization of COF-366-Co. (a) Crystal structure model of COF-366-Co. (b) Powder X-ray diffraction (PXRD) pattern of COF-366-Co.

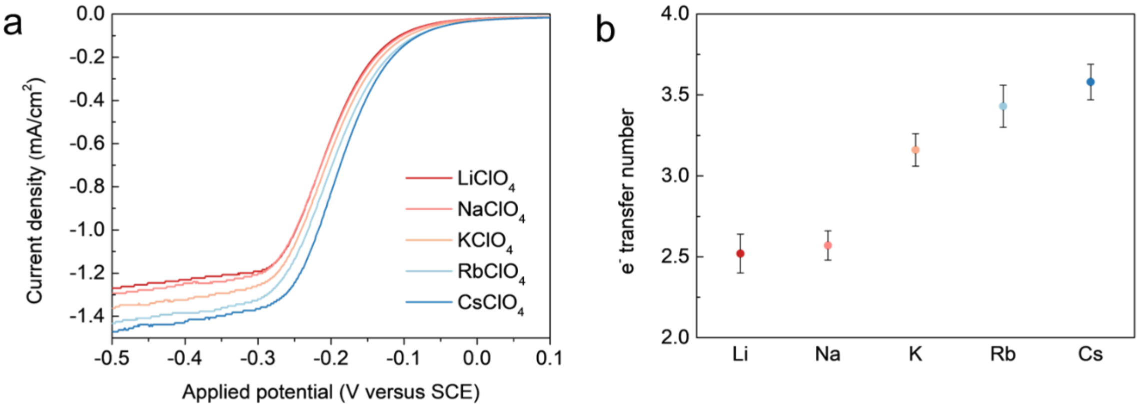

Figure S13. ORR catalyzed by COF-366-Co in various AMClO$_4$ electrolytes. (a) LSVs of ORR catalyzed by COF-366-Co in O_2-saturated 80 mM AMClO$_4$ electrolytes (AM= Li, Na, K, Rb, Cs). (b) Electron transfer number of ORR catalyzed by COF-366-Co in O_2-saturated 80 mM AMClO$_4$ electrolytes at -0.35 V.

Supplementary References

[1] Damjanovic A, Genshaw MA, Bockris JO'M. Distinction between intermediates produced in main and side electrodic reactions. *J Chem Phys* 1966; **45**: 4057–4059.

[2] Jin S, Van Neste A, Ghali E *et al.* New cathode materials for chlorate electrolysis. *J Electrochem Soc* 1997; **144**: 4272.

[3] Wroblowa HS, Yen-Chi-Pan, Razumney G. Electroreduction of oxygen: a new mechanistic criterion. *J electroanal chem interfacial electrochem* 1976; **69**: 195–201.

[4] Resasco J, Chen LD, Clark E *et al.* Promoter effects of alkali metal cations on the electrochemical reduction of carbon dioxide. *J Am Chem Soc* 2017; **139**: 11277–11287.
